# Modulating the Tumor Microenvironment to Enhance Tumor Nanomedicine Delivery

**DOI:** 10.3389/fphar.2017.00952

**Published:** 2017-12-22

**Authors:** Bo Zhang, Yu Hu, Zhiqing Pang

**Affiliations:** ^1^School of Pharmacy, Fudan University, Key Laboratory of Smart Drug Delivery, Ministry of Education, Shanghai, China; ^2^Institute of Hematology, Union Hospital, Tongji Medical College, Huazhong University of Science and Technology, Wuhan, China

**Keywords:** tumor microenvironment, nanomedicine, tumor nanomedicine delivery, interstitial fluid pressure, tumor perfusion, extracellular matrix

## Abstract

Nanomedicines including liposomes, micelles, and nanoparticles based on the enhanced permeability and retention (EPR) effect have become the mainstream for tumor treatment owing to their superiority over conventional anticancer agents. Advanced design of nanomedicine including active targeting nanomedicine, tumor-responsive nanomedicine, and optimization of physicochemical properties to enable highly effective delivery of nanomedicine to tumors has further improved their therapeutic benefits. However, these strategies still could not conquer the delivery barriers of a tumor microenvironment such as heterogeneous blood flow, dense extracellular matrix, abundant stroma cells, and high interstitial fluid pressure, which severely impaired vascular transport of nanomedicines, hindered their effective extravasation, and impeded their interstitial transport to realize uniform distribution inside tumors. Therefore, modulation of tumor microenvironment has now emerged as an important strategy to improve nanomedicine delivery to tumors. Here, we review the existing strategies and approaches for tumor microenvironment modulation to improve tumor perfusion for helping more nanomedicines to reach the tumor site, to facilitate nanomedicine extravasation for enhancing transvascular transport, and to improve interstitial transport for optimizing the distribution of nanomedicines. These strategies may provide an avenue for the development of new combination chemotherapeutic regimens and reassessment of previously suboptimal agents.

## Introduction

In recent times, nanomedicine delivery to tumors has attracted extensive attention in the field of tumor treatment (Allen and Cullis, 2004; Peer et al., 2007). The advantage of nanomedicines over free drugs is based on the enhanced permeability and retention (EPR) effect (Fang et al., 2011; Maeda, 2012). The fundamental characteristics of EPR physiology are highly permeable tumor vessels allowing the enhanced permeability (EP) of large particles including proteins, macromolecules, liposomes, micelles, and other particles large enough to avoid renal clearance, into the tumor interstitium combined with impaired lymphatic drainage limiting clearance and causing enhanced retention (ER) of those extravasated particles. Both features result from the rapid growth of a tumor and collapse of the existing blood and lymph vessels in the limited interstitial space (Leu et al., 2000; Dreher et al., 2006). With EPR effect as the main principle for passive targeting strategy, nanomedicine delivery to tumors has achieved success to varying degrees. However, the clinical benefits of the three EPR-based Food and Drug Administration (FAD)-approved nanomedicines including pegylated liposomal doxorubicin (Doxil/Caelyx), liposomal daunorubicin (DaunoXome), and nanoparticle albumin-bound paclitaxel (Abraxane) for the treatment of solid tumors were demonstrated to be only modest (O'Brien et al., 2004; Gradishar et al., 2005; Jain and Stylianopoulos, 2010), posing considerable challenges for the clinical translation of new nanomedicines. Accumulating evidence revealed that EPR-dependent drug delivery was always compromised by the tumor microenvironment including irregular vascular distribution, elevated tumor interstitial fluid pressure (IFP), poor blood flow, rich extracellular matrix (ECM) and abundant tumor stroma cells (Nichols and Bae, 2014). Delivery barriers posed by the tumor microenvironment are the main reasons responsible for the modest survival benefits of FDA-approved nanomedicines (Jain and Stylianopoulos, 2010).

The tumor microenvironment, as an important component of tumor tissues, functions as the soil for the seeds i.e., tumor cells to proliferate, differentiate, and promote tumor growth (Zhang et al., 2015, 2016a). The components of tumor microenvironment include the extracellular matrix (ECM) and different kinds of stromal cells such as tumor-associated fibroblasts (TAF), tumor-associated macrophages, and pericytes (Hanahan and Weinberg, 2011). As a pathologic condition, the tumor microenvironment is remarkably abnormal: tumor blood flow is low, perfusion is uneven in tumors, the tumor vessel permeability is highly heterogeneous, interstitial fluid pressure (IFP) is elevated, and a large number of active stromal cells and ECM are often dense and stiff.

Systemically administrated nanomedicines need to undergo a three-step process in solid tumors to achieve their therapeutic effect: vascular transportation to different areas of the tumor, trans-vascular transport across the vessel wall, and interstitial transport to reach the tumor cells (Wong et al., 2011). Delivery of nanomedicines differs markedly between tumors and normal tissues owing to structural differences. The abnormality in organization and structure of the tumor vasculature leads to heterogeneous blood flow, which directly influences the vascular transport of nanomedicines (Jain, 1994, 2005). Additionally, the vascular hyper-permeability and lack of functional lymphatic vessels inside tumors results in elevated IFP (Boucher et al., 1990), which not only compresses tumor vessels to aggravate the heterogeneous blood flow, but also reduces convective transport of nanomedicines (Jain, 1987). Besides, compression from proliferating tumor cells, stromal cells, and the ECM could also compress tumor vessels (Stylianopoulos et al., 2012). Furthermore, the dense ECM hinders interstitial diffusion of nanomedicines (Jain, 1987). When 90-nm liposomes, approximately the size of liposomal doxorubicin, an approved nanomedicine, was intravenously administered in tumor-bearing mice, these particles leaked out of tumor vessels but did not move far away from the vessel wall (Yuan et al., 1994a). Even directly intra-tumor injected 150-nm particles did not move far from the injection site (McKee et al., 2006). Altogether, the complex tumor microenvironment could negatively affect vascular transport, trans-vascular transport and interstitial transport of nanomedicine, and compromise nanomedicine delivery for tumor treatment.

To improve the therapeutic benefits of nanomedicine, different strategies including active targeting nanomedicine (Zhang et al., 2014a,b), tumor-responsive nanomedicine (Zhu et al., 2012; Huang et al., 2013), as well as optimization of the physiochemical parameters of nanomedicine such as size (Tong et al., 2013; Tang et al., 2014), charge (Han et al., 2015), and shape (Chauhan et al., 2011) have been developed. However, these methods rely on the advanced development of the nanomedicine itself, which could not conquer the above-mentioned delivery barriers of the tumor microenvironment (Chauhan and Jain, 2013). Accordingly, a modification of the tumor microenvironment was recognized as an important tool to improve tumor nanomedicine delivery (Jain and Stylianopoulos, 2010; Miao et al., 2015). In this review, in terms of the three processes including vascular transport, trans-vascular transport and interstitial transport that nanomedicines need to experience before reaching tumor cells and achieving therapeutic benefit, we tried to summarize different strategies of modulating tumor microenvironment to improve tumor nanomedicine delivery from the corresponding three aspects including improving tumor perfusion, facilitating nanomedicine extravasation, and enhancing interstitial transport of nanomedicine.

## Nanomedicine transport barriers from tumor microenvironment

**Figure 1:**
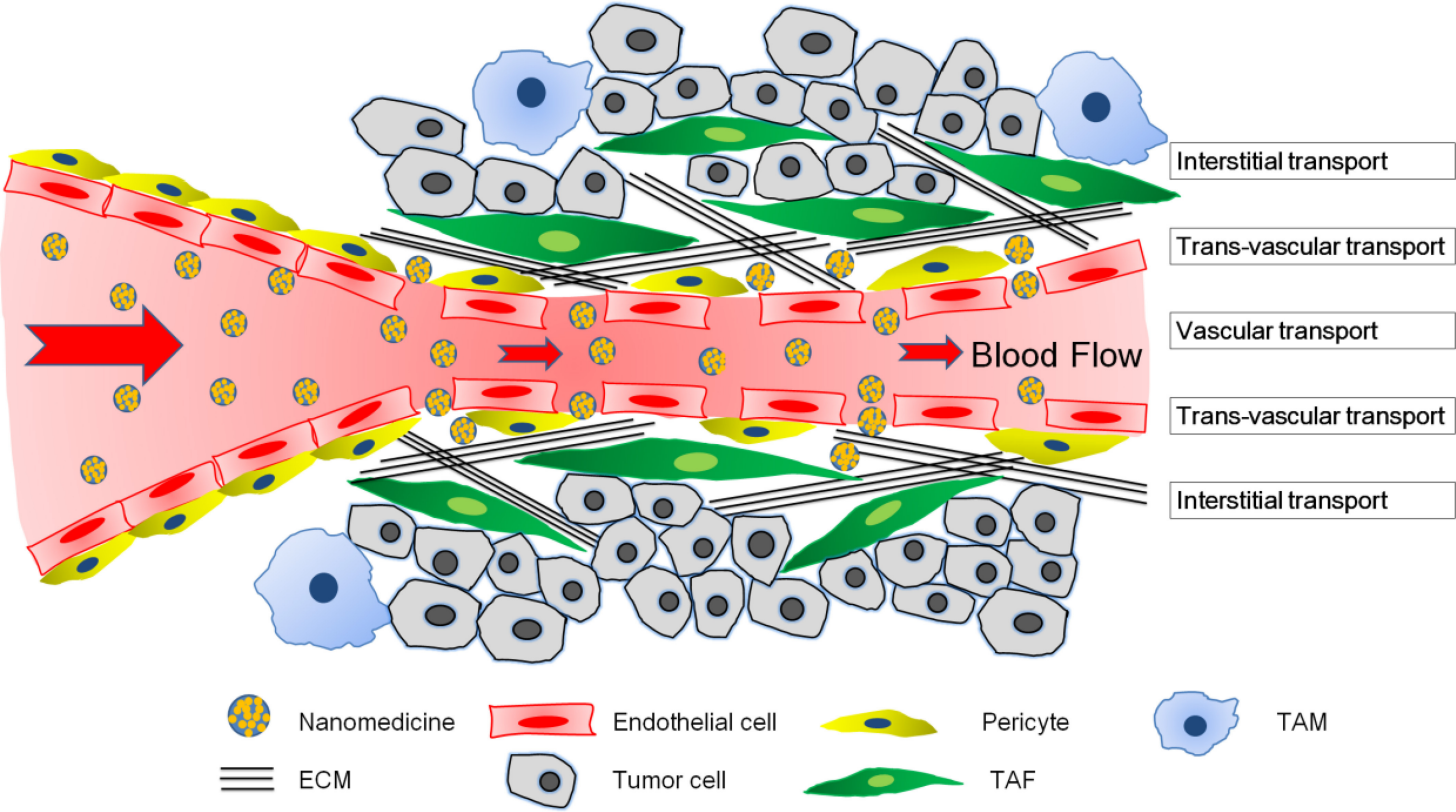
The transport barriers for tumor nanomedicine delivery imposed by a complicated tumor microenvironment including poor blood perfusion, IFP, dense ECM, and a large number of stromal cells. The nanomedicines have to cross the blood vessel walls, penetrate the extravascular space, and eventually reach tumor cells to exert their therapeutic effects.

The main transport barriers of a tumor microenvironment include abnormal tumor vasculature, elevated IFP, dense ECM, and stromal cells (Figure 1). These components varied with respect to different tumor types. To better understand and address the complexities of a tumor microenvironment, we used two representative models—highly permeable tumors and highly desmoplastic tumors (Stylianopoulos and Jain, 2013). Highly permeable tumors such as gliomas and melanomas are always rich in vessels and a small amount of pericytes, TAFs, and ECM, wherein tumor cells are in the vicinity of tumor vessels. On the other hand, highly desmoplastic tumors such as pancreatic cancers (Cabral et al., 2011), bladder cancers (Zhang et al., 2014), and some breast cancers (Stylianopoulos and Jain, 2013) are always hypovascular with numerous TAFs, a dense ECM, and high coverage rate of pericytes on the endothelium, such that the tumor cells are isolated into nests by TAF complexed with ECM and are a certain distance from the tumor vessels (Feig et al., 2012; Zhang et al., 2016c). Highly permeable and highly desmoplastic tumors were also referred to as tumors with tumor-vessels architecture and tumors with stroma-vessels architecture, respectively, in some reports (Smith et al., 2013; Miao et al., 2016).

### Abnormal tumor vasculature networks

In normal tissues, an exquisite counterbalance is achieved between the proangiogenic molecules such as VEGF and endogenous antiangiogenic molecules such as sVEGFR1 and thrombospondins (Carmeliet and Jain, 2000; Jain, 2003). In tumor tissues, however, the proangiogenic effect is abnormally upregulated and the pathological angiogenesis occurs in a disorganized manner. Compared to normal vessels, tumor vessels are highly irregular and chaotic in structure with wide endothelial gaps and a heterogeneous basement membrane (Carmeliet and Jain, 2011). The tortuous and leaky nature of tumor vessels contributes to compromised tumor blood flow, as the high tortuosity of tumor vessels results in an elevated geometric resistance that retards the blood flow (Sevick and Jain, 1989a). Furthermore, this sharp drop of blood flow due to the geometric resistance exerts a marked influence on the viscous resistance of blood in tumor vessels (Sevick and Jain, 1989b). Additionally, the leaky nature of tumor vessels allows the permeability of fluid into the interstitium. Fluid loss would increase the hematocrit of tumor blood to elevate its viscosity and further impede tumor blood flow (Sevick and Jain, 1989b; Netti et al., 1996; Sun et al., 2007). This combined effect of tortuosity of and leakage from tumor vessels compromises blood flow in brain tumors to one to three orders of magnitude, slower than that in the pial vessels surrounding normal tissues (Yuan et al., 1994b). Inefficient blood flow in tumors always leads to poor delivery of systemically administered drugs. Therefore, the tumor vascular network poses a major barrier in vascular transportation of nanomedicine, specifically highly permeable tumors with a variety of hyper permeable vessels.

### Elevated IFP

IFP is a type of stress exerted by fluids and is uniformly elevated throughout the tumor bulk in many tumors. Fluid flow is involved in three processes including flow along the tumor vessels, through the tumor interstitium, and drainage of excessive fluid by the lymphatic vessels (Jain et al., 2014).

Abnormalities in the tumor microenvironment concerning these three processes lead to elevated IFP. The leaky tumor vessels allow the extravasation of excessive fluid and plasma macromolecules into the tumor interstitium. In normal tissues, excessive fluid could be drained by an effective lymphatic network to maintain a balanced tissue interstitial pressure. However, lymphatic drainage in tumors do not function properly and hence, IFP is elevated in tumor tissues. Apart from abnormality in tumor blood vessels and lymphatic vessels, abnormal hydraulic conductivity is also a regulator of IFP, especially in highly desmoplastic tumors. The hydraulic conductivity depends on the volume fraction, surface charge, chemical composition, and organization of fibers in the tumor interstitial space (Levick, 1987; Stylianopoulos et al., 2008). Tumors abundant in collagen could display an order of magnitude lower in hydraulic conductivity than those with low collagen content (Netti et al., 2000). The negatively charged glycosaminoglycans could increase flow resistance because of their ability to trap water (Levick, 1987). Therefore, depletion of glycosaminoglycans with matrix metalloproteinases-1 and -8 increases the hydraulic conductivity and thus, the interstitial fluid velocity (Mok et al., 2007). Furthermore, a high density of tumor cells and stromal cells might reduce the interstitial space available for fluid flow, thereby increasing fluid resistance and IFP. In normal tissues, IFP is in the range of 0–3 mm Hg. However, experimental and human solid tumors exhibit high IFP, typically ranging from 5 to 40 mmHg, which may reach 75–130 mmHg in highly desmoplastic pancreatic tumors (Milosevic et al., 2004; Provenzano et al., 2012). Therefore, in many tumors, elevated IFP and reduced microvascular pressure (MVP) hinder nanomedicine delivery by the following mechanisms. First, as IFP and MVP also impose fluid stresses on vessel walls, elevated IFP could compress tumor vessels to cause blood stasis and/or vessel collapse to reduce the vascular transport of nanomedicine. Another consequence of high IFP is that interstitial fluid might escape from the tumor periphery into surrounding normal tissue, carrying not only nanomedicine to reduce the trans-vascular transport of nanomedicine but also growth factors or tumor cells to drive tumor metastasis or drug resistance (Chary and Jain, 1989; Netti et al., 1996). Finally, IFP might force the nanomedicine to extravasate by passive diffusion, instead of convention, a much faster transport process, which compromises the interstitial transport of nanomedicine especially larger nanomedicines. To conclude, elevated IFP inside tumors hinders vascular, trans-vascular, and interstitial transport of nanomedicines.

### ECM

The ECM is the non-cellular component widely present within all tissues and organs. ECM is mainly composed of two types of macromolecules: proteoglycans (PGs) and fibrous proteins (Järveläinen et al., 2009; Schaefer and Schaefer, 2010). PGs such as hyaluronan fill the majority of the extracellular interstitium of the tissue in the form of a hydrated gel. The fibrous ECM proteins include collagens, elastins, fibronectins, and laminins (Dequidt et al., 2007). ECM is a highly dynamic structure being constantly remodeled, either enzymatically or non-enzymatically, and its final components are controlled by a myriad of post-translational modifications. ECM is tissue-specific and the component varies greatly among different tissues including cancerous ones (Frantz et al., 2010).

Under normal conditions, the unique composition and structure of the ECM functions as a growth regulator. ECM and ECM-associated enzymes and growth factors regulate cell proliferation and differentiation, maintaining cell survival and dynamic homeostasis (Li et al., 2010). However, ECM is commonly deregulated and becomes disorganized in diseases such as cancer. Fibrosis due to excessive ECM production or limited ECM turnover occurs in many types of cancers. Especially in highly desmoplastic tumor such as pancreatic cancer and some breast cancers (Stylianopoulos and Jain, 2013), a dense ECM composed of collagen, hyaluronan, and fibronectin is always found (Feig et al., 2012; Zhang et al., 2016c). In contrast, tumors with abundant vessels always harbor a low level of ECM. Signaling pathways involved in ECM production included transforming growth factor-beta (TGF-β), Hedgehog (Hh) signaling, and platelet-derived growth factor (PDGF). ECM turnover is subjected to enzyme-mediated remolding including heparanase, cysteine proteases, 6-O-sulfatases, urokinase, and many matrix metalloproteinases (MMPs) (Egeblad et al., 2010; Lu et al., 2012). In highly vascularized and permeable tumors such as glioma and melanoma, ECM is always scarce.

The dense ECM in the tumor interstitium not only compressed tumor vasculature and reduced vascular transport of nanomedicine but also isolates tumor cells into nests within a certain distance from collapsed vessels and resists the free penetration and homogeneous distribution of nanomedicine in three main ways (Bailey et al., 2008; Miao et al., 2015). First, limited interstitial volume plus high stromal fraction and large matrix molecules result in a dense network (Padera et al., 2004), effectively reducing blood flow and limiting convection of nanomedicine. Second, the fibrillar structure, mesh size, and collagen thickness directly limit the diffusion of nanomedicine. The diffusion capacity is inversely related to the size of nanomedicine. Matrix mesh size ranges between 20 and 40 nm in solid tumors. Particles larger than the mesh size are completely prevented from diffusing through the ECM, those near the mesh size can be hindered to a certain extent, and only small particles can penetrate almost freely (Nichols and Bae, 2012). Third, the tortuous nature of the interstitial space poses an additional barrier for drugs of all size, because it elongates the diffusion path of the nanomedicine from blood vessels to tumor cells (Chauhan et al., 2009). The resistance of nanomedicine delivery from ECM mainly occurs in highly desmoplastic tumors. For highly permeable tumors with tumor-vessels architecture, the ECM is not as abundant, dense, and stiff as that in highly desmoplastic tumors, the and nanomedicine can penetrate throughout the tumor tissues much more easily after its extravasation from tumor vessels (Cabral et al., 2011). Unfortunately, the ECM is much more denser and thicker in human tumors than in mouse models (Miao and Huang, 2015). In conclusion, rich ECM in tumors resists vascular and interstitial transport of nanomedicine.

### Stromal cells

Stromal cells include TAF, tumor-associated macrophages (TAM), and pericytes. The origin of TAF is still debatable. TAF probably originated from resident tissue fibroblasts, bone marrow-derived mesenchymal stem cells, hematopoietic stem cells, epithelial cells (epithelial-mesenchymal transition; EMT), and endothelial cells (endothelial-mesenchymal transition; EndMT) (Shiga et al., 2015). It was now widely accepted that TAF significantly contributes to cancer progression (Brennen et al., 2012). TAF is abundant in highly desmoplastic tumors and produces large amounts of ECM to isolate tumor cells into a nest. TAF has been regarded as the major component of tumor stroma and contributes to the binding-site barrier for interstitial transport of nanomedicine (Miao et al., 2016). Large numbers of TAF associated with dense ECM also compress tumor vessels to compromise the vascular transport of nanomedicine. Besides, reports have shown that uptake of the anisamide ligand-modified nanomedicine by TAF was 7-fold higher than that of the other cells because of the different expression level of the sigma receptor between TAF and other cells (Miao et al., 2016).

TAM is the major cancer-related inflammatory cell primarily converted from monocytes that are closely associated with the prognosis of many cancer types. Other inflammatory cells include granulocytes, dendritic cells, and myeloid derived suppressor cells, which are also important constituents of the tumor microenvironment (Mocellin et al., 2001; Hu et al., 2016). When polarized toward the anti-inflammatory state by the tumor microenvironment, TAM promotes immune evasion and angiogenesis, thereby driving tumor growth (Cieslewicz et al., 2013). The off-target effect of nanomedicines is inevitable because of the phagocytic properties of inflammatory cells. Roode et al. showed that the association between TAM and NP were 4-fold greater than that of cancer cells despite TAM constituting only 1% of all cells in tumors (Roode et al., 2016). The off-target uptake of nanomedicine by stromal cells including TAF and TAM could certainly reduce the uptake of nanomedicine by tumor cells and therefore the therapeutic benefits.

Pericytes are another important type of stromal cells located mainly in the perivascular space, which also affect nanomedicine delivery. Neither leaky, immature vessels with little coverage nor over-mature vessels with high pericyte coverage are favorable for nanomedicine delivery. Excessively leaky vessels in highly vascularized tumors affect nanomedicine delivery mainly by compromising blood perfusion and thereby the vascular transport of nanomedicine (Stylianopoulos and Jain, 2013). In contrast, high pericyte coverage is always found in highly desmoplastic tumors, which reduce the endothelial gap and limit the trans-vascular transport of nanomedicine, especially for larger nanomedicine (Cabral et al., 2011).

## Strategies to modulate tumor microenvironment

In accordance with the three processes including vascular, trans-vascular and interstitial transport that nanomedicines need to experience before reaching tumor cells, strategies of modulating tumor microenvironment to improve nanomedicine delivery for tumor treatment can be divided into three categories: improving tumor perfusion, facilitating nanomedicine extravasation, and enhancing interstitial transport of nanomedicine (Table 1).

**Table 1:** Summary of tumor microenvironment modulation strategies for improving tumor nanomedicine delivery.

**Modulation strategies**	**Main working mechanism**	**Modulation agents**	**Tumors**	**References**
Improving tumor perfusion	Tumor vessel normalization by blocking tumor proangiogenic signaling	DC101 (VEGF mAb)	Mammary carcinoma, small cell lung carcinoma, glioblastoma multiforme, colon adenocarcinoma	Tong et al., 2004
		Bevacizumab (VEGF mAb)	Colon carcinoma, melanoma	Ellis, 2005; Turley et al., 2012
		SST0001 (Heparanase inhibitor)	Myeloma	Ritchie et al., 2011
		Rapamycin (mTOR signaling inhibition)	Melanoma	Guo et al., 2014
		Chloroquine (Notch 1 signaling inhibition)	Melanoma	Maes et al., 2014
		Dopamine (D2 receptors-angiopoietin 1 activation)	Prostate and colon tumor	Chakroborty et al., 2011
		Imatinib mesylate (PDGF signaling inhibition)	Lung carcinoma	Zhang et al., 2016d
	Tumor vessel dilation	BQ123 (ETA antagonist)	Colorectal carcinoma	Wang et al., 2017
		Captopril (hypotensor)	Glioma	Zhang et al., 2017a
Facilitating nanomedicine extravasation	Inflammatory mediators for enhancing vessel permeability	TNF- alpha	lymphoma and melanoma	Curnis et al., 2002; Seki et al., 2011
		Prostaglandin I2	Hepatocellular carcinoma	Tanaka et al., 2003
		VEGF	Glioma and colon carcinoma	Monsky et al., 1999
		Nitroglycerin (NG)	Sarcoma	Seki et al., 2009
	Pericyte depletion by inhibiting TGF signal pathway	A small-molecule TGF-β inhibitor, LY364947	Pancreatic cancer	Meng et al., 2013
		TGF- type I receptor (TR-I) inhibitor	Pancreatic cancer, gastric cancer	Kano et al., 2007
		ID11 (anti-TGF-β mAb)	Breast carcinoma	Liu et al., 2012
	Platelet depletion	Antiplatelet antibody R300	Breast cancer	Li et al., 2017
Enhancing interstitial transport	Direct ECM degradation	Matrix metalloproteinases-1 and−8	Sarcoma	Mok et al., 2007
		Hyaluronidase and hyaluronidase-loaded nanoparticles	Pancreatic cancer, breast cancer	Provenzano et al., 2012; Gong et al., 2016; Zhou et al., 2016
		PEGPH20 (PEGylated hyaluronidase)	Pancreatic cancer	Jacobetz et al., 2012; Hingorani et al., 2016
		rtPA	Lung cancer, melanoma	Zhang et al., 2016b; Kirtane et al., 2017
	ECM reduction by inhibiting TAF activity	IPI-926 (Hh inhibitor)	Pancreatic cancer	Olive et al., 2009
		Cyclopamine (Hh inhibitor)	Pancreatic cancer	Zhang et al., 2016c; Jiang et al., 2017
	TAF depletion or reprogramming	Losartan	Human breast, pancreatic, and skin tumors	Diop-Frimpong et al., 2011; Chauhan et al., 2013
		VDR ligand	Pancreatic cancer	Sherman et al., 2014
		ATAR	Pancreatic cancer	Froeling et al., 2011; Chronopoulos et al., 2016
		Quercetin nanoparticles downregulating Wnt16 expression	Bladder carcinoma	Hu et al., 2017

### Improving tumor perfusion

#### Tumor vasculature normalization

**Figure 2:**
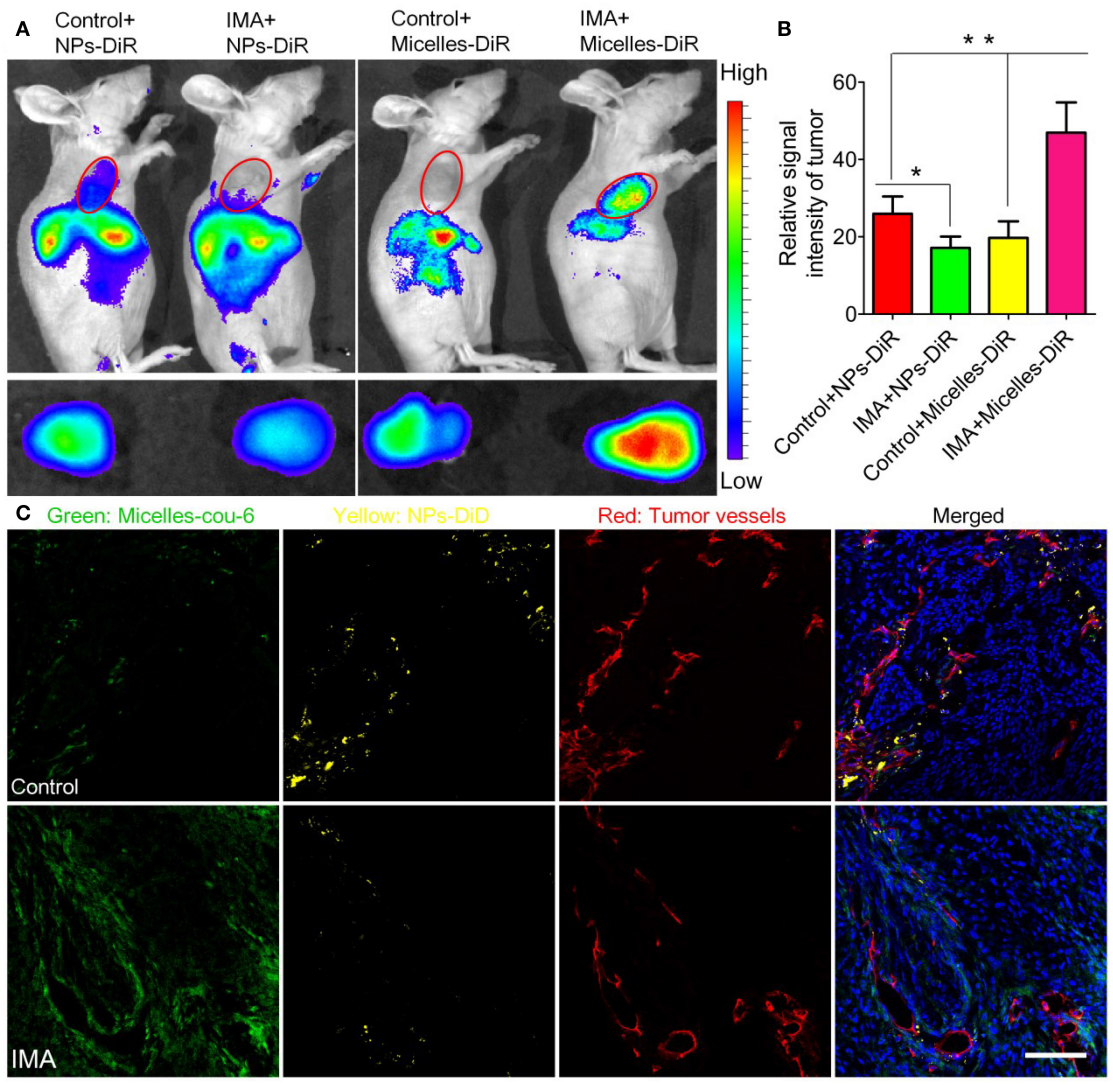
The effects of IMA treatment on tumor nanoparticle delivery. **(A)** *In vivo* fluorescence imaging of A549 xenograft-bearing mice (the upper row) treated with IMA or water as a control, *ex vivo* fluorescence imaging of their corresponding tumor xenografts (the lower row), and **(B)** the relative signal intensity of tumor tissue 24 h post the injection of DiR-labeled nanoparticles or micelles. ^*^p < 0.05, compared with Control+NP group. ^**^p < 0.01 compared with IMA+Micelles group. **(C)** *In vivo* distribution of micelles and nanoparticles in tumor slices from A549 tumor xenograft-bearing mouse models treated with IMA or water at 24 h after i.v. injection of a mixture of DiD-labeled nanoparticles and coumarin-6-labeled micelles. The oral dose of IMA was 50 mg/kg/d for 3 weeks. The dose of both coumarin-6 and DiD was 0.05 mg/kg. The bar indicated 100 μm. Reprinted from reference with permission by copyright holder, Zhiqing Pang.

The newly formed tumor vessels are always tortuous and leaky allowing the extravasation of nanomedicine but simultaneously increasing IFP, which prevents adequate and homogeneous blood flow and vascular transport of nanomedicine. To improve nanomedicine delivery for tumor treatment, normalization of vessels has emerged as an effective approach. Vessel normalization transforms the abnormal phenotype of tumor vessels into a phenotype that closely resembles that of fully functional normal vessels by repairing the basement membrane and increasing coverage rate of pericytes, and ultimately decreasing vessel leakiness. Optimizing the structure of tumor vessels could reduce the extravasation of excessive fluid and lower IFP, and then restore tumor blood flow, thereby improving vascular transport of nanomedicine. Many proangiogenic molecules including VEGF, fibroblast growth factor (FGF), and PDGF are overexpressed in tumors and involved in angiogenesis, which cause chaotic structural development in these newly formed tumor vessels (Goel et al., 2011). Therefore, strategies to block these proangiogenic signaling molecules were designed to repair tumor vessels. For example, VEGF inhibitors Bevacizumab, the FDA-approved antiangiogenic monoclonal antibody (mAb), capable of reverting abnormal structure of tumor vessels toward a more normal phenotype have been applied in the treatment of metastatic colorectal cancer (Salgaller, 2003; Ellis, 2005; Table 1), which were of high potential to improve nanomedicine delivery for tumor treatment. Moreover, some angiogenic signaling pathways such as mTOR signaling (Guo et al., 2014), Notch 1 signaling (Maes et al., 2014), and D2 receptors-angiopoietin 1 signaling (Chakroborty et al., 2011) involved in vessel normalization have also been modulated to improve nanomedicine delivery (Table 1). In our previous research, it was also shown imatinib mesylate (IMA) could normalize the tumor vessels of A549 tumors by inhibiting platelet-derived growth factor signaling pathway (Zhang et al., 2016d). Interestedly, IMA treatment could significantly reduce the accumulation of nanoparticles (NPs) around 110 nm but enhanced the accumulation of micelles around 23 nm. Furthermore, IMA treatment limited the distribution of NPs inside tumors but increased that of micelles with a more homogeneous pattern (Figure 2). Finally, the anti-tumor efficacy study displayed that IMA pretreatment could significantly increase the therapeutic effects of paclitaxel-loaded micelles. As tumor vessel normalization minimized endothelial gap, it could prevent tumor cells shedding into tumor vessels, and reduce the possible tumor metastasis to a certain degree.

To utilize vessel normalization strategy to improve nanomedicine delivery for tumor treatment, four concerns should be kept in mind. First, the strategy can only improve the delivery of small molecular weight drugs or relatively smaller nanomedicines ranging from 20 to 40 nm, but reduces the delivery of large nanomedicines around 100 nm as it decreases the endothelial gap of tumor vessels (Chauhan et al., 2012; Zhang et al., 2016d, 2017b). Second, the normalization is transient and the followed nanomedicine should be applied in the normalization window. Third, a judicious dose of vascular normalizer is highly recommended to prevent excessive pruning of tumor vessels, which might impair vascular efficiency and thus the delivery of concurrent therapy (Huang et al., 2012; Jain, 2013). Fourth, as vasculatures are always severely compressed in highly desmoplastic tumors and are refractory to vasculature normalizers (Smith et al., 2013), this strategy might only be used in tumors that are highly permeable but not highly desmoplastic, or at least combined with other strategies capable of reopening compressed vessels.

#### Tumor vessel dilation

Vasoconstrictive endothelin-1 (ET1) and its receptor ETA via which ET-1 mediates vasoconstriction are both abundant in tumor tissues for maintaining the contractile tone of tumor vessels. The expression level of ET1 and ETA in tumor vessels was 13-fold and 5-folder higher than that of size-matched normal vessels, respectively (Sonveaux et al., 2004). BQ123, a selective antagonist against ETA could inhibit ET1-ETA signaling, induce tumor vessel dilation, and trigger a tumor-specific increase in blood flow. The blood flow improvement induced by BQ123 improved the delivery of free drugs to tumors despite an increase in IFP (Martinive et al., 2006). In addition, it was also demonstrated that BQ123 could increase the delivery of photothermal nanomedicine around 100 nm for effective photothermal therapy of tumors (Wang et al., 2017). Some inflammation factors such as bradykinin capable of dilating vessels could also directly increase tumor perfusion. In our previous study, it was shown captopril, a widely used hypotensor in clinics, could dilate tumor blood vessels by increasing bradykinin expression and even increase tumor vessel permeability to enhance nanomedicine delivery for tumor therapy (Zhang et al., 2017a).

### Facilitating nanomedicine extravasation

#### Inflammatory mediators for enhanced permeability

Inflammatory mediators such as TNFα (Seki et al., 2011), prostaglandin analogs (Tanaka et al., 2003), VEGF (Monsky et al., 1999), and nitric oxide (NO) donors (Seki et al., 2009), capable of enhancing vascular permeability, have been utilized to increase nanomedicine accumulation in tumors up to 2–6-fold higher than that of the control group (Table 1). Apart from vascular permeability enhancement, vasodilatation and blood-flow improvement by usage of inflammatory mediators were also involved in improving nanomedicine delivery for tumors. However, a series of effects of inflammatory mediators mentioned above could also lead to elevated IFP against nanomedicine delivery. Thus, accumulation of nanomedicine in tumors comprehensively depends on these factors. As inflammation might potentially promote cancer development (Atsumi et al., 2014), local application (Seki et al., 2011) or targeted delivery of inflammatory mediators to the tumor site should be adopted.

#### Pericyte depletion

In highly desmoplastic tumor, the coverage rate of pericytes on endothelium was about 70%, much higher than highly permeable tumors, which significantly limit the transvascular movement of nanomedicine into tumor interstium. Therefore, strategies by using low dose of a TGF-β inhibitor, LY364947 was developed to reduce the pericyte coverage of endothelium and increase size gaps between endothelium to increase therapeutic benefits of gemcitabine-loaded liposomes for pancreatic cancer (Cabral et al., 2011; Meng et al., 2013) and Doxil for diffuse-type gastric cancer (Kano et al., 2007).

#### Platelet depletion

It is well known that platelets contribute a lot to hemostasis. Apart from its role in thrombus formation, platelets are highly involved in tumor progression and metastasis. In addition, it could also support tumor vascular homeostasis and protect the integrity of tumor vessels (Kisucka et al., 2006; Ho-Tin-Noé et al., 2009). Studies showed that platelets reduction induced bleeding in the tumor site and increased leakiness of tumor vasculature. Therefore, platelets reduction in thrombocytopenic mice increased efficacy of chemotherapy for breast cancer (Demers et al., 2011). To avoid potential bleeding in normal organs caused by low platelet counts, a recent study by Li et al. designed a tumor microenvironment-responsive nanoparticle capable of delivering antiplatelet antibody R300 to selectively deplete platelet in tumor tissues, therefore augmenting vascular permeability and improving nanomedicine delivery for tumors (Li et al., 2017). Platelet depletion represented as a promising approach to augment transvascular delivery of nanomedicine to tumors.

#### Physical stimulus

Radiation can improve tumor-targeted delivery of nanomedicine (Davies Cde et al., 2004; Giustini et al., 2012). Some possible mechanisms are as follows: First, radiation could upregulate the level of vascular endothelial growth factor (VEGF) by activating hypoxia inducible factor 1 (HIF1) (Moeller et al., 2004; Stapleton et al., 2016) or via multiple mitogen-activated protein kinase dependent pathways (Park et al., 2001) to increase the permeability of tumor vessels (Kobayashi et al., 2004). Results showed that the tumor vessels' permeability of magnetic resonance imaging-contrast agent with the molecular weight above 200 kDa was increased by 32.8% after irradiation (10 Gy). In addition, radiation can rapidly kill the sensitive tumor cells. The reduced cell density helped to alleviate compression stress from tumor cells, reopen collapsed vessels, and therefore increase tumor blood flow (Nagano et al., 2008; Khawar et al., 2015). The effect of radiation on tumors is complex and dose-, time-, and tumor-type dependent (Garcia-Barros et al., 2003; Fuks and Kolesnick, 2005; Kioi et al., 2010). Milosevic's recent review provides further evidence of the same (Stapleton et al., 2016).

Kong et al. pioneered the use of mild hyperthermia (HT) for nanomedicine extravasation into tumor tissues by improved vascular permeability (Kong et al., 2000, 2001). Studies further demonstrated that mild HT could also help improve tumor perfusion and reduce IFP (Sen et al., 2011; Winslow et al., 2015), probably via creation of vascular fenestrations and perturbation of the vascular endothelium (Kirui et al., 2015), and thus allow deep nanomedicine penetration throughout the tumors, rather than perivascular accumulation (Li et al., 2013). However, there was no direct evidence to demonstrate the pore size change of tumor vessels after HT treatment. The extravasation depth and intensity in tumor interstitium was considered to vary greatly among different types of tumors, which depends on the pattern of the endothelial lining and the intrinsic property of the surrounding tumor microenvironment (Eberhard et al., 2000), such as structure of the interstitial matrix. Apart from tumors with a certain vascular component, highly desmoplastic tumors could also respond well to mild HT treatment (Kirui et al., 2014, 2015). The temperature is a crucial element in the heating method, wherein results showed that 41–43°C was appropriate, because a very high temperature might damage the endothelial lining of tumor vessels and induce coagulation response. Thrombin formation could choke the vessels and compromise nanomedicine delivery. Alternatively, insufficient temperature might exert a very minimal effect on the tumor vessel endothelium to increase the endothelial gap (von Maltzahn et al., 2011; Li et al., 2013).

Ultrasound has been used to improve nanomedicine delivery for tumors by both mechanical and HT effect (Goins et al., 2016). For mechanical effects, many reports showed that gas-filled bubbles could be used to transiently produce pores in blood vessels (Durymanov et al., 2015) or cell membranes (sonoporation) (Yoon et al., 2014; Ma et al., 2016), through which nanomedicines of different types can effectively extravasate tumor vessels or enter into tumor cells (Thakkar et al., 2013), therefore achieving improved delivery of nanomedicine (Rapoport et al., 2013, 2015). Besides, ultrasound also produces heat at an acoustic intensity and in a time-dependent manner. Recently, Frazier used magnetic resonance imaging-guided, high-intensity focused ultrasound (HIFU) to produce a spatially uniform 43°C heating pattern in a xenograft tumor model and improved the accumulation of Evans blue dye in heated tumors to nearly 2-fold higher than in unheated tumors (Frazier et al., 2016).

### Improving interstitial transport of nanomedicine

#### ECM disruption strategies

Dense ECM always resists free penetration of nanomedicine throughout tumor tissues to reach tumor cells. Therefore, modification of ECM has been extensively explored to improve the delivery and distribution of nanomedicine in tumor tissues. The ECM modification strategy includes direct ECM disruption and reduction of ECM synthesis by inhibiting TAF activity.

Several studies have shown that different kinds of enzymes directly degrade the components of ECM such as collagen and hyaluronic acid and can improve the delivery of nanomedicine (Table 1). For instance, collagenase-coated nanomedicine could penetrate deeper into the core of *in vitro* tumor spheroids than control ones (Goodman et al., 2007; Cui et al., 2013). Besides, enzymatic digestion of collagen and decorin facilitates >10-fold increase in the diffusion of macromolecular dextran into tumor tissue, supporting matrix degradation as a useful tool to improve macromolecule distribution (Magzoub et al., 2008). In another study, intravenous injection of collagenase-1 into xenograft-bearing mice models increased the accumulation and gene expression of lipoplex in tumors by 1.5- and 2-fold, respectively, further confirming collagen digestion to be a useful strategy to improve nanomedicine delivery (Kato et al., 2012). Hyaluronan, or hyaluronic acid, a large linear glycosaminoglycan, composed of repeating N-acetyl glucosamine and glucuronic acid units, was also a crucial component of ECM (Provenzano et al., 2012), which was found abundant in non-small cell lung cancer (NSCLC), prostate, pancreatic, and breast cancers. Hyaluronidase was shown to induce a 4-fold increase in the distribution of liposomal doxorubicin in a human osteosarcoma xenograft model (Eikenes et al., 2005). A phase 1b trial of docetaxel combining PEGPH20 in metastatic refractory NSCLC has been completed (NCT02346370) with results pending (Wong et al., 2017). In addition, a nanomedicine combining PEGylated hyaluronidase (PEGPH20) to improve the efficiency of chemotherapeutics for hyaluronan-high pancreatic cancer is currently in phase 3 clinical trial (NCT02715804) (Provenzano et al., 2012; Wong et al., 2017). Lysyl oxidase (LOX) is a key element in the crosslinking of collagen and increasing the stiffness of collagen fibers (Egeblad et al., 2010; Kanapathipillai et al., 2012). LOX-activity inhibition has proven successful in preventing ECM remodeling and stiffening (Levental et al., 2009; Barry-Hamilton et al., 2010), which may overcome the deregulated ECM barrier for nanomedicine delivery (Khawar et al., 2015).

**Figure 3:**
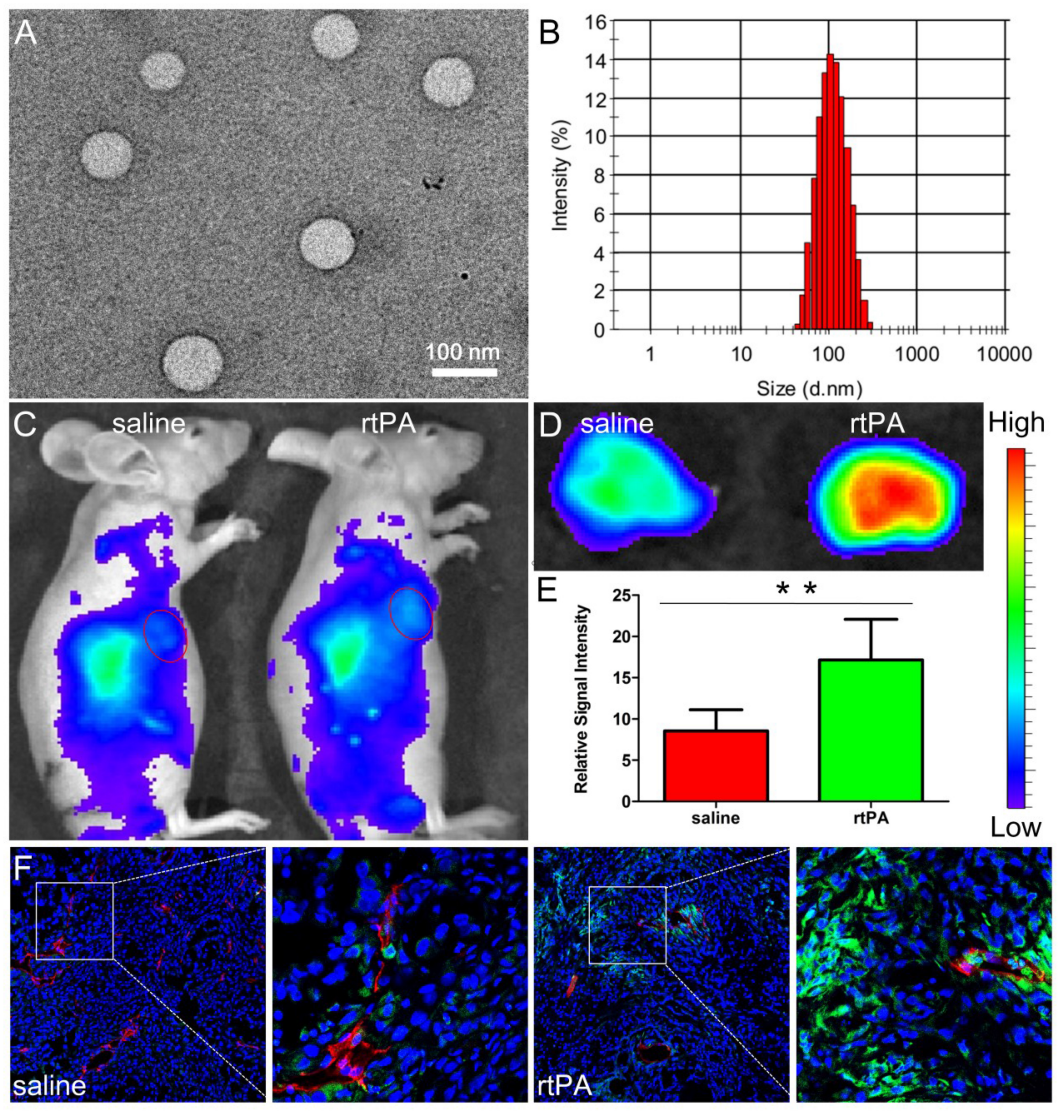
Characterizations of NPs and effects of rtPA treatment on tumor nanoparticle delivery. TEM photograph **(A)** and size distribution **(B)** of NPs. Bar: 100 nm. *In vivo* **(C)** and *ex vivo* imaging **(D,E)** of A549 xenograft-bearing mice treated with 2 weeks of rtPA (25 mg/kg/d) or saline 24 h after the injection of DiR-labeled NPs. ^**^p < 0.01 rtPA vs. saline group. *In vivo* distribution of NPs in tumor tissues. **(F)** Original magnification: 120 ×. Reprinted from reference with permission, Copyright Elsevier, 2016.

It was noteworthy that ECM disruption was seldom used to increase nanomedicine delivery in highly vascularized tumors, which might be the relative lack of ECM in these tumors. However, our group found that fibrin, a kind of ECM component was rich in tumors harboring rich tumor vessels (Dvorak, 1986). The reason might be due to leakage of coagulation factors from circulation to tumor tissues and the high express level of tissue factor on tumor cells, both of which together contribute to local coagulation response in tumor tissues (Dvorak et al., 1985; Liu et al., 2011). As the end product of coagulation response, fibrin is mostly covalently cross-linked in tumor interstitium as an important component of tumor ECM (Dvorak, 1986; Pilch et al., 2006) and is mainly located in the vicinity tumor vessels (Nakahara et al., 2006), a distinct distribution pattern totally different from that of other components of matrix such as collagen and hyaluronic acid, which are always extensively distributed throughout tumor tissues. The special distribution pattern of fibrin was demonstrated to compress tumor vessels nearby, which reduce blood flow and compromise nanomedicine delivery for tumors. Moreover, as fibrin is always covalently cross-linked near tumor vessels, the penetration of nanomedicines in the tumor interstitium could also be hindered. Treatment with rtPA, a clinically widely used drug, at a dose of 25 mg/kg for 2 weeks, could safely and successfully deplete fibrin deposition, reopen compressed tumor vessels, reduce erythrocytes retention in tumor vessels, improve tumor blood flow, and further enhance the accumulation and penetration of nanoparticles (Figure 3; Zhang et al., 2016b).

However, systematic treatment with ECM disruptors such as collagenase, hyaluronidase, and rtPA may cause damage to healthy tissues, and site-specific action might be safer for clinical transformation. Therefore, tumor-specific degradation of ECM was achieved by coating nanomedicines with specific ECM enzymes (Zhou et al., 2016) or PEGlated ECM enzymes (Hingorani et al., 2016). Zhou et al. showed that hyaluronidase modified on the surface of nanoparticles was more effective than free hyaluronidase to help facilitate nanoparticle diffusion and achieved better therapeutic benefits (Zhou et al., 2016). Reports also showed that the dose of hyaluronidase seemed critical, because high doses of hyaluronidase may collapse water-swelling cage structures of hyaluronan rendering the ECM more viscous and less permeable, thereby reducing the diffusion coefficient of nanomedicine (Eikenes et al., 2010). In another research, Bromelain, a crude enzymatic complex purified from pineapple stems that belongs to the peptidase papain family, was decorated to mesoporous silica nanoparticles (Br-MSN), which showed an increased ability to digest and diffuse in tumor ECM *in vitro* and *in vivo* (Parodi et al., 2014).

TAF was mainly responsible for ECM production. Resting fibroblasts were transformed to TAF by cancer-derived growth factors such as TGF-β, Hedgehog moiety (Olive et al., 2009; Stylianopoulos et al., 2012), and PDGF (Olson and Hanahan, 2009). This trans-differentiation process of TAF is always characterized by the encoding of ECM-associated components such as collagens, hyaluronan, fibronectin, and MMPs (Cirri and Chiarugi, 2011). Therefore, ECM deregulation can be realized by blocking the growth factors involved in signaling for TAF stimulation. Taking TGF-β-associated signaling as an example, antibodies or other agents capable of blocking TGF-β signaling have proven to inhibit collagen synthesis and enhance nanomedicine delivery in xenograft models. The TGF-β neutralizing antibody ID11 improved tumor tissue delivery of Doxil and obtained better control of mammary carcinoma in xenograft models (Liu et al., 2012). Recently, our group used cyclopamine, a naturally occurring steroidal alkaloid, to inhibit the Hedgehog signaling pathway which contributes a lot to ECM production in pancreatic carcinoma by acting on the Smoothened (SMO) receptor (Heretsch et al., 2010). Compared with the control group, cyclopamine treatment successfully disrupt ECM in in pancreatic cancer, increased functional vessels about 2 folds at a dose of 50 mg/kg for 3 weeks, and significantly improved the accumulation (by a 2.6-fold) and penetration of nanoparticles in tumor tissues (Zhang et al., 2016c).

ECM modulation suits nanomedicine regardless of their size, because ECM modification could reopen compressed vasculatures to improve tumor blood flow and decrease the hindrance of nanomedicine penetrating ECM to reach tumor cells (Chauhan and Jain, 2013). Generally speaking, this strategy most benefits the delivery of larger nanomedicines (Cabral et al., 2011; Jacobetz et al., 2012), as they are more hindered by the ECM (Pluen et al., 2001).

#### Stromal cell reprogramming or depletion strategies

Apart from dense ECM, desmoplastic tumors always harbor a high density of stromal cells, among which TAF has been regarded as the major component of tumoral stroma as a potential therapeutic target for nanomedicine delivery. TAF depletion can improve the interstitial transport and distribution of nanomedicine by optimization of the tumor interstitium. Quercetin nanoparticles, capable of suppressing Wnt16 expression could reduce the number of TAF and improve nanomedicine delivery to bladder carcinoma (Hu et al., 2017). Inspired by the close association between cyclooxygenase-2 (COX-2) and tumor-associated angiogenesis, as well as tumor matrix formation, our group explored the tumor microenvironment modulation effect of celecoxib, a special COX-2 inhibitor widely used in clinics. Very interestingly, oral celecoxib treatment at a dose of 200 mg/kg for 2 weeks could successfully normalized the tumor microenvironment, including tumor-associated fibroblast depletion, fibronectin bundle disruption, tumor vessel normalization, and tumor perfusion improvement. Furthermore, it also significantly enhanced the *in vivo* accumulation and deep penetration of 22-nm micelles rather than 100-nm nanoparticles in tumor tissues and improved the therapeutic efficacy of paclitaxel-loaded micelles in tumor xenograft-bearing mouse models (Zhang et al., 2017b).

Although TAF depletion could undoubtedly modify the tumor microenvironment to improve nanomedicine delivery, recent studies have also indicated that direct TAF depletion might drive tumor metastasis and progression (Özdemir et al., 2014), suggesting a paradoxical effect of TAF depletion. One explanation for this paradoxical effect is that the TAF-depleting strategy runs the risk of eliminating the key element needed for tissue homeostasis (Miao et al., 2015). To avoid this paradox, an alternative approach is to transform activated TAF to a dormant form. Losartan is a antihypertensive agent with anti-fibrosis properties. Research has shown that losartan treatment reprogramed TAF and reduced the collagen and hyaluronan content in desmoplastic models of human breast and pancreatic tumors in mice and improved the distribution and therapeutic effects of systematic administered Doxil (Diop-Frimpong et al., 2011; Chauhan et al., 2013). The antifibrotic effect of losartan was associated with reduced number of activated TAFs and therefore decreased expression of downstream profibrotic factors, such as connective tissue growth factor (CTGF), TGF-β1, and ET-1 (Chauhan et al., 2013), which led to an ongoing clinical trial of losartan combined with chemotherapy in pancreatic tumors (NCT01821729). Vitamin D receptor (VDR) and Wnt-β-Catenin signaling pathway was upregulated in pancreatic stellate cell (PSC), a form of TAF in PDA (Omary et al., 2007). Other studies showed that the VDR ligand and all-trans retinoic acid (ATRA) can act through VDR or Wnt-β-Catenin signaling pathway to reprogram PSC to the quiescent state to reduce the fibrotic content in tumor interstitium (Froeling et al., 2011; Sherman et al., 2014; Chronopoulos et al., 2016), which is promising for the second-wave nanomedicine therapy.

TAMs are prominent components and critical modulators of the tumor microenvironment and contribute to tumor development, invasion, and metastases. Evidences have shown TAMs protect tumor cells from chemotherapy and suppress the immune response of cytotoxic T cells (Jinushi et al., 2011), highlighting the essential of targeting TAMs for cancer treatment. TAM depletion could decrease off-target uptake of nanomedicine by TAMs and thus increase drug delivery to tumor cells. However, the effect of selective TAM depletion on nanomedicine delivery for tumor was seldom reported, and TAM depletion might be a new strategy for tumor microenvironment modulation to enhance tumor nanomedicine delivery.

## Future perspectives

Nanomedicine drug delivery system has attracted extensive attention in the field of tumor treatment. The complex tumor microenvironment including structural abnormalities in tumor vessels, dense ECM structure, and high density of stromal cells as well as physicochemical environment such as elevated IFP pose barriers and compromise the delivery of nanomedicines. In this review, we summarized these barriers and provided strategies to overcome them for improved nanomedicine delivery.

However, some aspects deserve special attention. First, tumors could be generally divided into two types such as those with abundant permeable but uncompressed vessels and tumors with dense ECM and large amount of TAF. More precise classification of tumor microenvironment-like tumor cells might be urgently needed for precise tumor nanomedicine delivery. We need to adopt different strategies according to the characteristics of the tumor microenvironment. For example, tumor vessel normalization is more effective for tumors with abundant, highly permeable but not compressed vessels, but not so appropriate for highly desmoplastic tumors. ECM disruption strategy demonstrates promising prospects to enhance nanomedicine delivery for tumors with abundant ECM even in clinical trials. As for tumors with a certain amount of both vessels and ECM, strategies capable of modulating vessels and ECM should be combined to obtain an optimal effect. In addition, it is important to study new animal models capable of quantitative analysis of parameters involved in the tumor microenvironment, such as IFP to quantify the negative contribution of tumor microenvironment parameters to tumor nanomedicine delivery and help develop corresponding strategies. Furthermore, the tumor microenvironment is too complex and one strategy might have multiple modulation effects on the tumor microenvironment. With respect to ECM disruption, it not only disrupt tumor ECM to optimize interstitial transport of nanomedicines, but also alleviate compression for tumor vessels to improve tumor perfusion to bring more nanomedicines to the tumor site. Another good illustration is the tumor vessel normalization strategy. Tumor vessel normalization could repair tumor vessels structure, which could improve tumor perfusion and reduce IFP to increase the delivery of small nanomedicine around 20–40 nm. However, it inversely compromised the delivery of larger nanomedicine around 100 nm because of the size reduction of endothelial gaps.

Therefore, nanomedicines with suitable qualities should be combined with approaches modulating the tumor microenvironment to overcome nanomedicine transport barriers that the advanced design of nanomedicines cannot conquer. With these in mind, we believe nanomedicines of the future could be far more effective than those available at present.

## Author contributions

ZP and YH conceived the principal idea and revised the manuscripts. BZ and ZP co-wrote the manuscript.

### Conflict of interest statement

The authors declare that the research was conducted in the absence of any commercial or financial relationships that could be construed as a potential conflict of interest.
